# Intelligent yield estimation for tomato crop using SegNet with VGG19 architecture

**DOI:** 10.1038/s41598-022-17840-6

**Published:** 2022-08-10

**Authors:** Prabhakar Maheswari, Purushothamman Raja, Vinh Truong Hoang

**Affiliations:** 1 School of Mechanical Engineering, SASTRA Deemed University, 613 401, Thanjavur, India; 2 Ho Chi Minh City Open University, Ho Chi Minh City, 70000 Vietnam

**Keywords:** Electrical and electronic engineering, Computer science

## Abstract

Yield estimation (YE) of the crop is one of the main tasks in fruit management and marketing. Based on the results of YE, the farmers can make a better decision on the harvesting period, prevention strategies for crop disease, subsequent follow-up for cultivation practice, etc. In the current scenario, crop YE is performed manually, which has many limitations such as the requirement of experts for the bigger fields, subjective decisions and a more time-consuming process. To overcome these issues, an intelligent YE system was proposed which detects, localizes and counts the number of tomatoes in the field using SegNet with VGG19 (a deep learning-based semantic segmentation architecture). The dataset of 672 images was given as an input to the SegNet with VGG19 architecture for training. It extracts features corresponding to the tomato in each layer and detection was performed based on the feature score. The results were compared against the other semantic segmentation architectures such as U-Net and SegNet with VGG16. The proposed method performed better and unveiled reasonable results. For testing the trained model, a case study was conducted in the real tomato field at Manapparai village, Trichy, India. The proposed method portrayed the test precision, recall and F1-score values of 89.7%, 72.55% and 80.22%, respectively along with reasonable localization capability for tomatoes.

## Introduction

Agriculture plays a pivotal role in economic growth all over the world as it reported 4% Gross Domestic Product (GDP) in developed countries and more than 25% GDP in developing countries for the year 2018 (https://www.worldbank.org). Specifically, the fundamental source for the economic development of India is agriculture as it contributes to 40% income of the nation. There is always a demand for (new) intelligent systems in this field for enhancing productivity in order to get sustainable economic growth. In this regard, precision agriculture contributes more to tackling the challenges in the agricultural domain. The contribution is immense to meet the demand of the rising population by enhancing the production through modern farming techniques^1^. YE of fruits is one of the important areas in the agricultural domain as it predicts the quantity of production. Traditionally, YE was performed manually by employing experts for their opinion (which is subjective based on their skill), a time-consuming process for bigger fields, etc. Hence, there is a need to develop an intelligent system to estimate the yield of fruits in an agricultural field^2^.

Deep Learning (DL) is the subfield of Machine Learning (ML), which is the main method used in Artificial Intelligence (AI). In ML, the source objects (like images, videos and audios) cannot be given directly to the learning models for training i.e., the features extracted from the source objects are the input to the learning models. DL is the advanced ML method where there is no requirement for feature extraction as it is hierarchical learning (i.e., the architecture itself extracts the abstract features at each level)^1,3^. With the advancement in technologies, (for example, using cameras on mobile phones) a good amount of datasets can be collected from an agricultural field. DL provides encouraging results through smart handling of big data due to the (cheap) widespread availability of excellent computational resources like Graphics Processing Unit (GPU)^4^.

For better object (i.e., fruit in our work) detection and localization, semantic segmentation is one of the techniques used in image processing. It provides complete information regarding a particular scene. Through semantic segmentation, instead of predicting the whole image to a particular class (this is the scenario in a normal classification task), every pixel is labeled to a specific class. In the past, various works have been performed for estimating the yield of fruits using computer vision employing semantic segmentation techniques^5–8^. Before the DL era, these traditional works mainly focused on the colour, texture, shape, size, etc., as the features in order to identify and segment the fruits. The hand-engineered features are used for classifying each pixel of an image, which is time-consuming as well as accurate localization of objects is difficult to obtain. After the DL era, there is a tremendous improvement in classification related problems. By combining DL with semantic segmentation, both classifications as well as localization of objects can be achieved better than the above said traditional techniques.

Recently various DL methods combined with semantic segmentation based pre-trained architectures namely, U-Net^9^, atrous convolution^10^, faster Region based Convolutional Neural Network (R-CNN)^11^, etc., have been developed for the image-based object recognition tasks. SegNet^12^ is one such pre-trained architecture used for the semantic segmentation of objects. The YE of various fruits namely apple, mango, citrus, etc., have been explored in the past using different pre-trained DL-based semantic segmentation architectures. Bargoti and Underwood^13^ developed an image segmentation framework to detect and count the fruits in an apple orchard. Two feature learning-based algorithms were used namely multi-scale multi-layer perceptron and Convolutional Neural Network (CNN). Watershed segmentation algorithm and circular Hough transform were used to segment the fruit pixels. The multi-scale and multi-layer perceptron network revealed the F1-score of 75.1% and the CNN revealed the F1-score of 79.1%. This method is simple and faster but the results can be improved for enhanced fruit detection in challenging environments.

Liu et al.^14^ proposed a method for counting the orange and apple fruits in an array of images. Video frames were tracked and deep segmentation combined with 3D localization was used for obtaining accurate results. Training and tracking of frame sequencing were done by Fully Convolutional Network (FCN) and Hungarian algorithm, respectively. Fruit size estimation along with 3D localization were carried out using the structure from the motion algorithm. The L1 loss, error mean and standard deviation were used to measure the counting capability of the proposed method. For the orange dataset, L1 loss of 203, error mean of − 0.2% and standard deviation of 26.3% were obtained. For the apple dataset, L1 loss of 322, error mean of 3.3% and standard deviation of 4.1% were obtained. Both results were obtained for the corrected model (i.e., after performing 3D localization). The work reported better detection capability in challenging practical conditions but the architecture can be improved for the 3D localization of fruits in order to reduce the error furthermore. Bresilla et al.^15^ proposed a method using single-shot detectors for detecting the fruits from the tree images. Apple and pear fruits were used to test an experimental architecture of a single-shot detector. The training was performed with 5,000 images and obtained a test accuracy of more than 90%. Few modifications like changes in the input grid and removing the selected layers were performed on the primary model of You Only Look Once (YOLO). It provided the advantage of an increase in processing speed at the expense of loss in accuracy. Table 1 shows the different literature related to DL-based semantic segmentation used for various fruit detection and localization tasks.

**Table 1:** Literature related to various fruit YE using DL-based semantic segmentation.

Author	Fruit	Description	Results
Apolo-Apolo et al.^19^	Citrus	Detection, counting and size estimation was done using faster R-CNN. The images were captured from the 20 sample trees of the citrus grove using a UAV	Precision, Recall and F1-score of 96%, 94% and 95% were obtained, respectively
Chen et al.^20^		Detection and localization were performed using an improved YOLO version4 (YOLOv4). Citrus tree images were captured using Kinect V2 camera and the architecture detects the small fruits accurately from the complex background	Accuracy varies from 92.89 to 96.04%
Kestur et al.^21^	Mango	Detection was performed using CNN with a pixel-based prediction method. The training and testing were performed from the patches of 11,096 and 1,500 which were obtained from the original images of 40 and 4, respectively	Accuracy and F1-score of 73.6% and 84.4%, were obtained, respectively
Koirala et al.^22^		Detection and counting were done using MangoYOLO, a modified version of YOLOv3 and tiny YOLOv2. The training, validation and test datasets of 1,300, 130 and 300 images were used, respectively	The overall F1-score is 89%
Borianne et al.^23^		Detection was done using faster R-CNN. Mango cultivars namely Kent, Keitt and Boucodiekhal were detected using the confidence and the non-maximal suppression threshold of 0.7 and 0.25, respectively	F1-score is 90%
Fu et al.^24^	Kiwi	Detection was done using faster R-CNN with Zeiler Fergus network (ZFnet). The 2,100 sub-images (of size 784 × 784 pixels) were obtained from the 700 original images (of size 2352 × 1568 pixels) captured from the field	The overall accuracy is 92.3%
Bargoti and Underwood^25^	Mango, apple and almond	Detection and counting were done by faster R-CNN. The tree images for the three categories of fruits were captured using Digital Single-Lens Reflex (DSLR) camera with the resolution varying from 2 to 17 megapixels	The overall F1-score is greater than 90%
Sa et al.^26^	Sweet pepper	Detection was performed using multi-modal faster R-CNN. The images were taken by two modalities namely RGB and NIR (Near-Infra Red) images. Early and late fusion methods were employed to combine the modalities	F1-score is 83.8%

Tomato is one of the important horticultural crops cultivated globally and the yearly production is around 180.64 million metric tons in 2019 (http://www.fao.org/faostat/en/#home). Due to the rich content of nutrition, it is taken in many forms like raw fruit, sauce, salad, etc. There is always a high demand for tomato productivity as it is predominantly used in most of the traditional cuisines. Some of the DL-based semantic segmentation architectures were explored for the intelligent YE of tomatoes. Liu et al.^16^ proposed an algorithm in order to detect the mature tomatoes using a Support Vector Machine (SVM) classifier by extracting the features using the Histogram of Oriented Gradients (HOG) descriptor. The false detections were removed using the false color removal method. The results revealed the test precision, recall and F1-score of 94.41%, 90% and 92.15%, respectively. The work revealed better performance for separate (i.e., single) fruit detection having ripened tomatoes. But the accuracy can be improved when the area of blockage is greater than 50%.

Yamamoto et al.^17^ developed an approach to detect the tomatoes using a ML method by extracting the color, shape and size features. In this work, pixel-based segmentation followed by blob-based segmentation was performed for an individual detection of tomato. Hence, two sets of training datasets were used. The work obtained the precision and recall values of 88% and 80%, respectively during testing phase. The work needs two sets of the dataset for both pixel-based and blob-based segmentation. Hence preprocessing for the images to be trained becomes a time-consuming task. Afonso et al.^18^ recommended a method for the detection of ripe and unripe tomatoes using mask R-CNN. They have used various pre-trained DL architectures (such as ResNet50, ResNet101 and ResNext101) as the backbone network for training the mask R-CNN. The precision and recall metrics were calculated for three sets of overlap IoU threshold values of 25%, 50% and 75%. For all the architectures, the metrics (i.e., precision, recall and F1-score) were high for an overlap threshold of 25% and low for an overlap threshold of 75% for single class fruit detection (i.e., both green and red pixels were labeled as tomato class). For the double class fruit detection (i.e., green pixels were labeled as green tomato class and red pixels were labeled as red tomato class), the metrics were high for an overlap threshold of 50%. The work revealed better detection of tomatoes using simple inexpensive cameras. However, the results can be improved by adding depth information as an additional input layer.

Lawal 2021^27^ developed a framework for modified YOLOv3 that detects and localizes the tomato in the complex environment such as occlusion, illumination variation, etc. Therefore, the tomato images were collected with a high degree of variability for training the architecture. Three different modified versions of YOLOv3 were used in this work. All three versions employed the different activation functions along with the front detection layer and evaluated the detection performance. Three versions of modified YOLOv3 achieved an average precision of 98.3%, 99.3% and 99.5% along with the detection time of 48 ms, 44 ms and 52 ms, respectively. This work provided an increase in precision, recall, F1-score and average precision by using an activation function of Mish. The literature related to tomato fruit YE performed using various DL-based semantic segmentation architectures is shown in Table 2.

**Table 2:** Literature for the YE of tomato fruit using DL-based semantic segmentation.

Author	Fruit	Description	Results
Mu et al.^28^	Tomato	Detection, counting and size estimation were performed using faster R-CNN with ResNet101. The architecture was already trained with the public dataset i.e., Common Objects in Context (COCO) dataset and the transfer learning technique are employed for tomato detection. This method provided an appreciable prediction for tomato yield estimation. However, longer training time and counting issues if the fruits are considerably shaded by the leaves can be further improved	Precision is 87.83% for IoU threshold greater than 0.5 and the coefficient of determination value R^2^ for tomato count is 0.87
Liu et al.^29^		Detection and localization were done by the modified version of YOLOv3 called YOLO-Tomato. Better localization of tomatoes was achieved by replacing the traditional rectangular bounding box with a circular	Precision, recall and F1-score of 94.75%, 93.09% and 93.91%, were obtained, respectively
Rahnemoonfar and Sheppardy^30^		Detection and counting were done using a modified Inception-ResNet-A module. The architecture was trained using the synthetic (tomato) image dataset whereas tested using the real data. The architecture was efficient even in challenging conditions such as shadow, some degree of overlap, etc. However, it couldn’t count the green fruits as the training was done only for ripe and half-ripe fruits	Training and testing accuracy of 93% and 91% were obtained, respectively

Very few approaches were proposed for estimating the tomato fruit yield using a DL-based semantic segmentation approach^28,30^. It needs to be further explored with other architectures. SegNet is one of the DL-based semantic segmentation architectures which provides better object detection and localization with reduced computational complexity since only the max-pooling indices are transferred to the decoder (instead of the entire feature map) for preserving the spatial resolution. Hence, the objective of this work is to develop an intelligent YE system in order to count the number of tomatoes in an agricultural field using SegNet with VGG19 architecture.

To improve the various sectors of the agriculture field, precision agriculture employs advanced techniques for developing intelligent systems. One of the emerging techniques called DL provides state of the art results in many fields. Using this technique, the proposed intelligent system for tomato yield estimation has been developed. In this work, the original pre-trained SegNet which uses the first 13 layers of VGG16 is modified by adding 3 more layers (each on the encoder and decoder side) by using the base network of VGG19. The modified architecture uses the first 16 layers of VGG19 and removes the last three fully connected layers. This fine-tuning makes the architecture suitable for better feature extraction from the objects. This customized architecture detects and counts the tomato in an open field and to the best of our knowledge, it is the pioneering (or the first) work for tomato fruit detection using SegNet with VGG19.

## Materials and methods

### Tomato YE using SegNet with VGG19

Various steps involved for tomato YE using SegNet are collection, annotation and augmentation of images, implementation using SegNet with VGG19 and performance evaluation. These steps are discussed in the subsequent sections.

### Data collection

The images were captured using four Intel RealScene cameras fixed on the trolley at the tomato greenhouse in the Netherlands. The dataset of 123 RGB images^18^ used for this work is acquired from the publicly available dataset under a creative common license. (https://data.4tu.nl/articles/dataset/Rob2Pheno_Annotated_Tomato_Image_Dataset/13173422), (https://creativecommons.org/licenses/by/4.0/). All the images were taken at a distance of 5 m from the tomato crop. To avoid illumination variations (due to sunlight and cloud), the images were captured at night. All the 123 RGB images are 720 × 1280 pixels in size and these images are then transferred to the workspace of the windows10 processor with Intel® Core™ i7, 8th generation, 32 GB RAM for further processing.

### Data annotation

**Figure 1:**
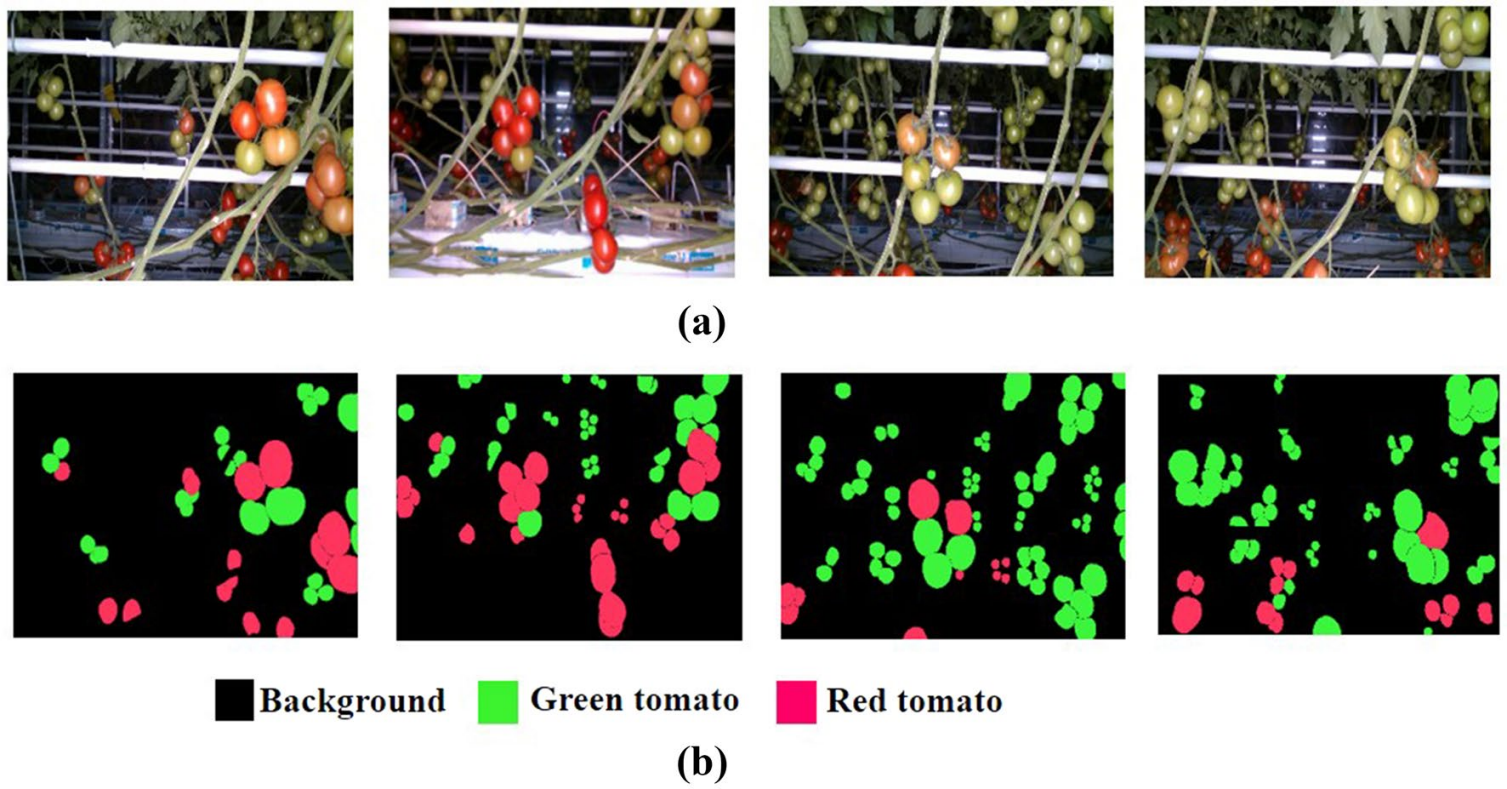
Image data before and after pre-processing (**a**) sample original images from the tomato dataset (Afonso et al.^18^), (**b**) sample ground-truth images.

For training the SegNet architecture, two types of the dataset are required, the original and its corresponding ground-truth dataset. The annotated version of original images is called ground-truth images. Computer Vision Annotation Tool (CVAT) was used for annotation (https://cvat.org). Three labels have been chosen namely ripe tomatoes (for red tomato pixels), unripe tomatoes (for green tomato pixels) and the background (for the remaining pixels). Figure 1 shows the sample original and its corresponding ground-truth images.

### Data augmentation

The original images are augmented in order to increase the size of the dataset for better generalization of the results. A lower dataset provides better results only in the training phase and generally fails to predict unobserved (i.e., test) data. It will lead to the lower performance of the proposed model. To avoid this issue, augmentation is one of the essential pre-processing steps to increase the size of the dataset thereby improving the generalization capability of DL architecture. In this work, augmentation is performed by applying transformations such as translation (i.e., ± 5 units in both x and y directions) and rotation (i.e., ± 10°) in the collected images. After the successful augmentation, the final size of the augmented dataset used for training the SegNet architecture is (comprising of) 672 images.

### Implementation using SegNet with VGG19

SegNet is one of the DL-based semantic segmentation architectures used in various object detection tasks. It uses the concept of upsampling during decoding for better localization of objects. Upsampling operation is performed by obtaining pooling indices from an encoder. In earlier DL-based semantic segmentation architectures, it was performed by transferring an entire feature map from an encoder. This results in an extra memory whereas SegNet reduced the memory requirements^31^.

After pre-processing of images, training was performed using SegNet architecture. The first 16 layers of VGG19^32^ are used as an encoder for feature extraction. The same 16 layers are also used in the decoder, but the layer of max-pooling is being replaced by an upsampling, for better spatial resolution^12^***.***

**Figure 2:**
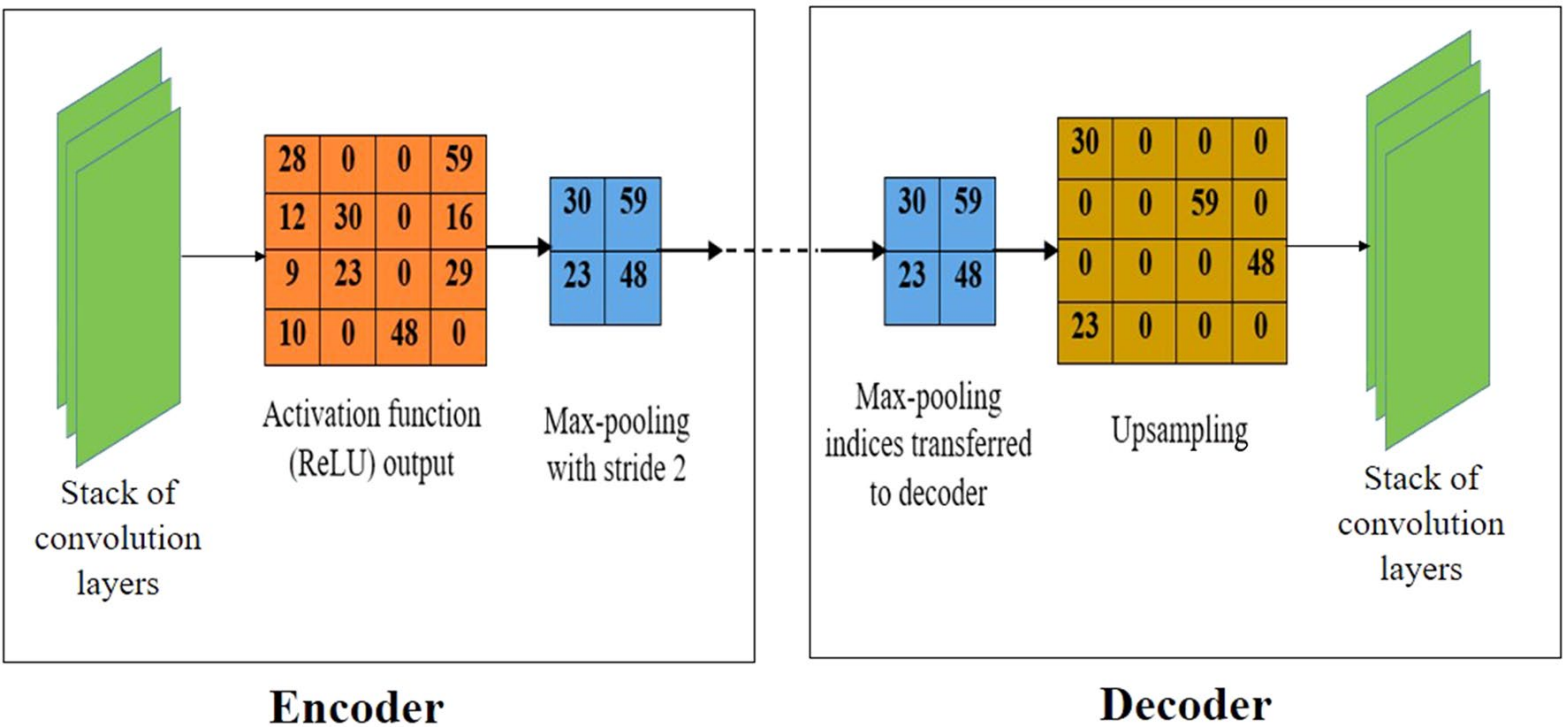
Upsampling operation in the proposed method of SegNet with VGG19.

Each pixel of an image is segmented into a certain class (i.e., red tomato, green tomato or background) using the architecture of SegNet. The convolution layer performs an element-wise dot product followed by a non-linear operation in which the Rectified Linear Unit (ReLU) is used as the activation function. This will allow only the positive values of input and all other values were changed to zero at the output. Then downsampling (i.e., max-pooling) is performed in order to reduce the spatial dimension as well as the number of computations, thereby reducing the network complexity. Upsampling followed by convolution operation performed on the decoder side results in dense pixel-wise prediction. Figure 2 shows the upsampling operation performed in the proposed method of SegNet with VGG19. In the encoder, after applying an activation function (ReLU), the feature map is subjected to the downsampling by performing max-pooling in order to reduce the computation. Pooling outputs the maximum value with a stride of 2. In the SegNet with VGG19, the max-pooling indices are only taken to the decoder for recovering the spatial resolution (instead of an entire feature map) thereby reducing the computational complexity. Using this SegNet with VGG19 architecture, the training has been performed (for detecting the tomatoes) which is described in the next section.

#### Training process

**Figure 3:**
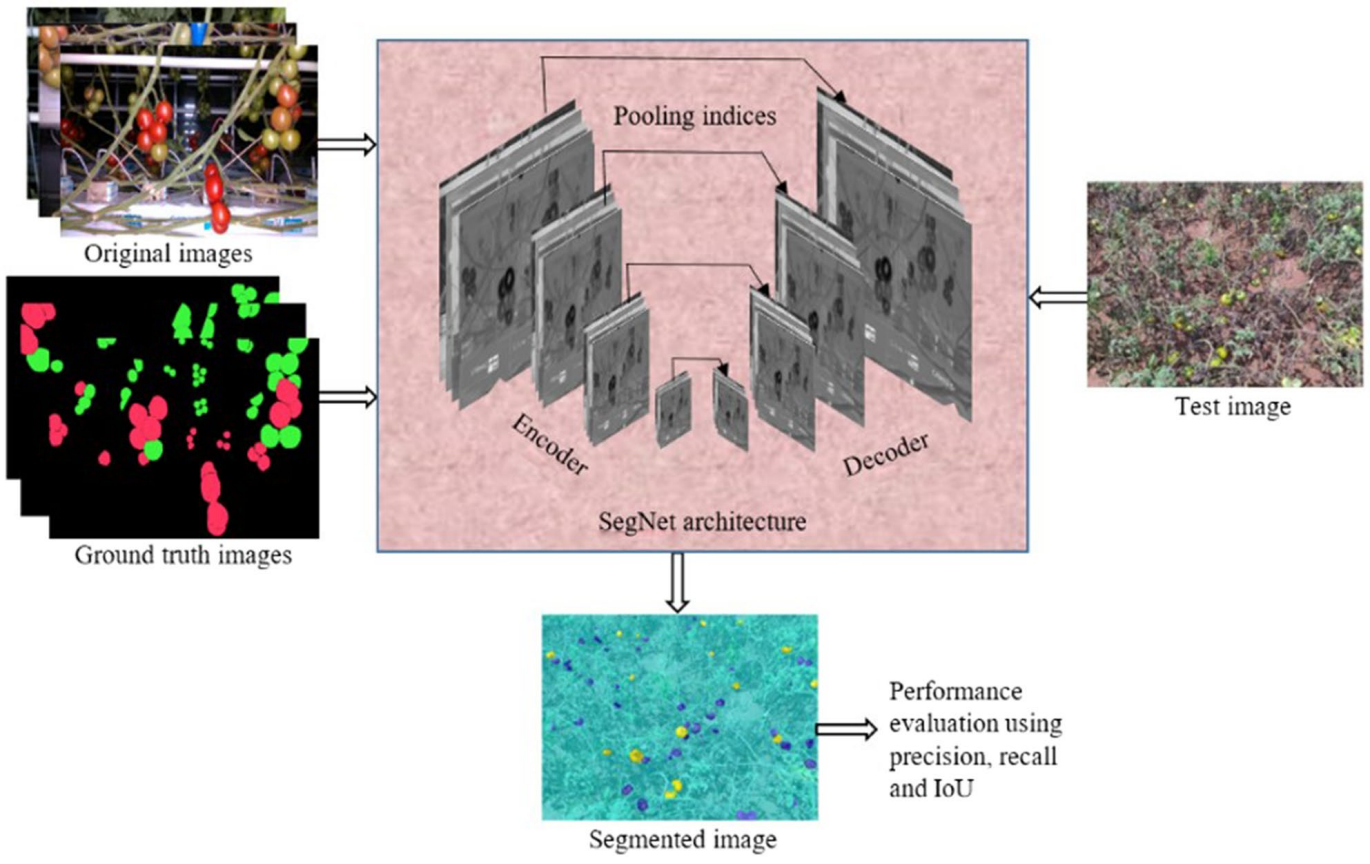
Training the tomato dataset using SegNet with VGG19.

The training process is depicted in Fig. 3. The original and labeled (i,e., ground-truth) images are the inputs given to the SegNet with VGG19 architecture.

From an encoder side, the stack of convolutional layers extracts features from the images at each level by convolving the matrices of the input images using the weighted filter. Initially, random weights are chosen and then using the Stochastic Gradient Descent (SGD) back propagation algorithm, the weights are optimized by nullifying the errors. At one point, the training will be stopped with a reduced error. The encoder performs transposed convolution where the max-pooling indices (present in the pooling layers) are transferred to the decoder.

From the decoder side, before convolution, upsampling of the feature map is done by obtaining the max-pooling indices from an encoder. While doing so, the spatial resolution lost during the operation of downsampling (during encoding) will be recovered. For a better estimation of yield, the location of tomatoes in each image needs to be preserved. This is achieved by upsampling. Further, the stack of convolutional layers (employed in the decoder) is used to obtain the dense feature map. Finally, based on the score map, pixel-wise prediction is performed to identify whether the specific pixel belongs to the assigned three categories i.e., red tomato (ripe), green tomato (unripe) or background (leaf, branch, stem, etc.). Then the training parameters were chosen by trial and error method for optimizing the weight and bias of SegNet architecture. The training parameters used for tuning the network are as follows:

- Optimization: SGD
- Initial learning rate: 0.001
- Mini batch size: 8
- Number of epochs: 100
- Iteration per epoch: 67
- Maximum iteration: 6700
- Regularization: L2
- Learning rate drop factor: 0.3.

### Performance evaluation

In order to assess the detection, various performance metrics such as precision, recall, Jaccard Index (JI) or Intersection over Union (IoU), F1-score, etc., are used to evaluate the SegNet architecture^33^. These performance metrics are defined in terms of True Positive (TP), False Positive (FP), False Negative (FN) and True Negative (TN). Precision in Eq. (1) measures the true positives within the positive observations which in turn indicates the reliability of an architecture. Recall in Eq. (2) indicates the detection capability of true positives of an architecture. IoU in Eq. (3) measures the ratio between intersection and union of ground-truth and predicted pixels (of the segmented output) for each class of an image. F1-score in Eq. (4) provides the measure for obtaining the harmonic mean of both recall and precision. It is the single score that gives the balanced results between recall and precision values.

1 $$Precision = \frac{TP}{{TP\, + \,FP}}$$

2 $$Recall = \frac{TP}{{TP\, + \,FN}}$$

3 $$IoU = \frac{TP}{{TP\, + \,FP\, + \,FN}}$$

4 $$F1{\text{-}}score = 2\left[ {\frac{{Precision* Recall}}{{Precision + Recall}}} \right]$$

where TP—Actual and predicted class label is tomato. TN—Actual and predicted class label is background. FP—Actual label is background but predicted as the tomato. FN—Actual label is tomato but predicted as background.

**Figure 4:**
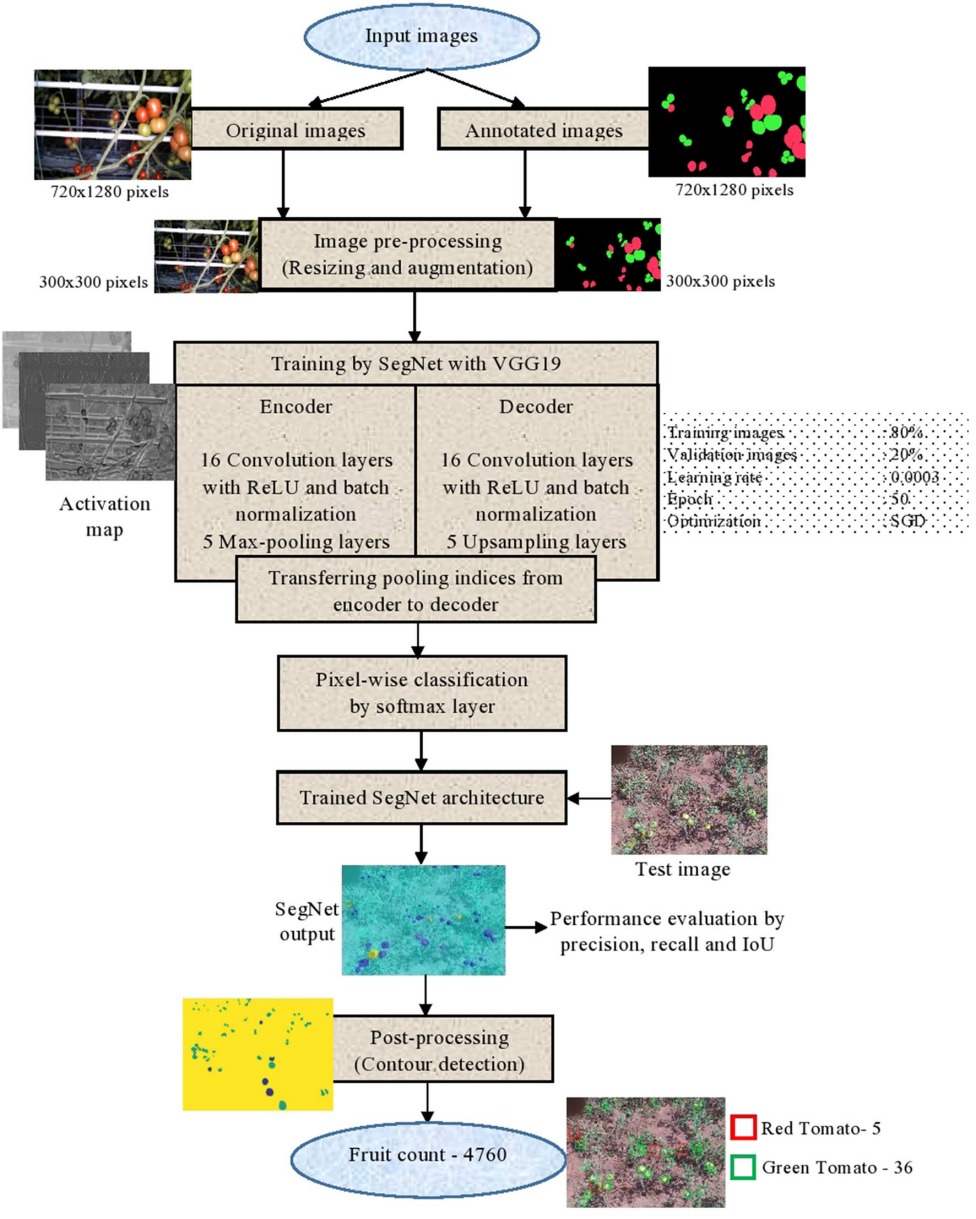
Overall flow of the algorithm of the proposed method.

The overall flow of the algorithm is depicted in the flow chart as shown in Fig. 4.

## Results and discussion

MATLAB 2021a and the GPU of NVIDIA Geforce GTX 1080 were used for coding and processing the data, respectively. This GPU version has the Compute Unified Device Architecture (CUDA) cores of 2,560 and the standard memory of 8 GB GDDR5X with a memory speed of 10Gbps.

### Performance metrics

**Figure 5:**
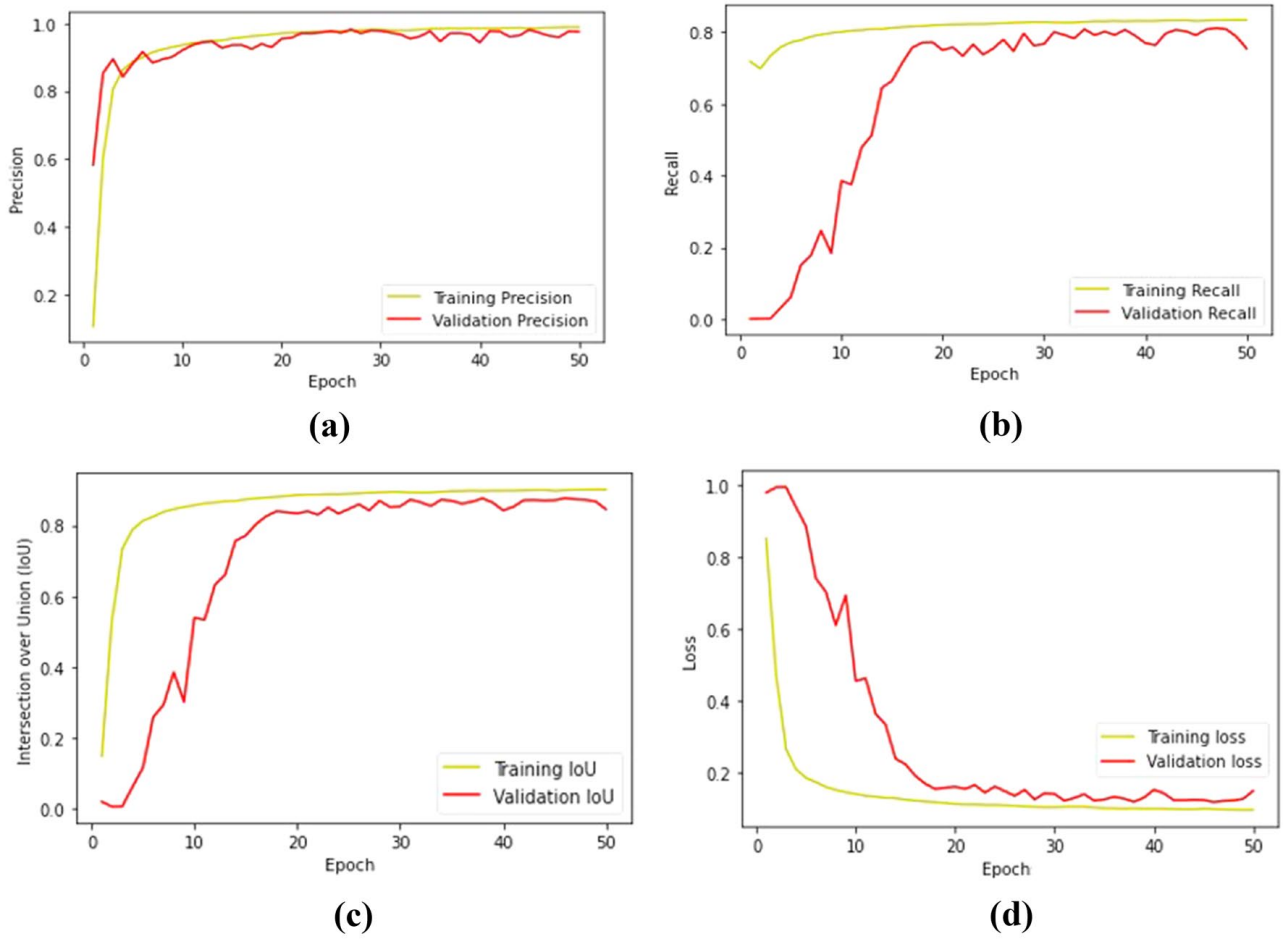
Performance metrics graph for training and validation (**a**) training and validation for precision, (**b**) training and validation for recall, (**c**) training and validation for IoU, (**d**) training and validation for loss.

The splitting of the dataset was performed with an 80:20 ratio, i.e., 80% of the images are trained to employ SegNet architecture using the training parameters as in “Training process” section. The remaining 20% of the images are validated after training. The performance of the proposed method was evaluated for both the training and validation dataset using the metrics as in “Performance evaluation” section. Figure 5 shows the training and validation graphs plotted for the performance metrics of precision, recall, IoU and loss for every epoch.

### Comparison of results

The training and validation results inferred that almost all the tomatoes were detected from a given input image using SegNet with VGG19 architecture. For instance, if the fruits are not correctly localized, the fruit picking system may not move to the next target location correctly. The primary task for any automatic fruit harvesting system depends upon the localization of fruits.

Therefore, in order to assess the localization capability of the proposed method, the comparison was performed with other architectures namely SegNet with VGG16 and U-Net. VGG16 is one of the pre-trained architectures of CNN^32^ used for image based recognition tasks. It is also used as the backbone network for developing the encoder and decoder of the SegNet architecture. U-Net is one of the recently developed DL-based semantic segmentation architectures which provides better results for objection detection^9^. Here the feature extraction is performed by contraction path and spatial resolution is retained by expanding path. The same dataset (used in our proposed work) is fed to the SegNet with VGG16 and U-Net. The comparison details of all the architectures in terms of precision, recall, IoU and F1-score are presented in Table 3.

**Table 3:** Performance comparison with other architectures.

Architecture	Precision in %		Recall in %		IoU in %		F1-score in %	
	Training	Validation	Training	Validation	Training	Validation	Training	Validation
U-Net	90.29	85.4	80.26	70.55	88.2	80.1	84.98	77.28
SegNet with VGG16	94.25	90.3	81.22	72.44	90.0	82.9	87.25	80.39
SegNet with VGG19	99.05	97.62	84.82	75.37	91.16	84.57	91.38	85.06

From Table 3, it was observed that the proposed SegNet with VGG19 demonstrated higher validation values in terms of precision, IoU and F1-score of 97.62%, 84.57% and 85.06%, respectively against SegNet with VGG16 and U-Net architectures. Further, SegNet with VGG19 obtained the validation value in terms of recall was 75.37%, but SegNet with VGG16 and U-Net achieved 72.44% and 70.55%, respectively indicating a similar trend. For SegNet with VGG19, analysis shows that the reduced value of recall (i.e., 75.37% when compared to precision and IoU) is due to the cases of a few false negatives (i.e., actual tomato pixel is wrongly predicted as background pixel by the architecture). For SegNet with VGG16 and U-Net, the cause for comparatively higher false negatives is due to the various challenging conditions such as the features of background pixels similar to the tomato pixels, illumination variations, etc.

For testing unobserved data, a case study was performed at the real tomato field which is explained in the next “Case study” section.

### Case study

In order to test the proposed model for the estimation of yield, a tomato field has been chosen at Manapparai village (longitude of 78° 26′ 49″ E and latitude of 10° 37′ 22″ N), Trichy. The selected field has an area of about ¼acre and the variety chosen is *Novo84.* From the chosen field, the selected sample size is 33 × 16ft., which is approximately equal to $$\tfrac{1}{\begin{subarray}{l} 80 \\ \end{subarray} }$$ acres. The sample selection is based on the size and shape of plots used for the study of field crops by Huddleston et al.^34^.

A total of 65 images were captured from the sample location, one week before harvesting using a 16 MP *Oneplus A6000* mobile camera. Each image has a width and height of 4608 × 3456 pixels, respectively. The images are then subjected to pre-processing (as discussed in “Materials and methods” section) and then fed to the SegNet architecture with VGG19. The past year (manual) YE data was collected from the farmers for comparison with the predicted results of the proposed method. Using the optimal parameters, the captured (real) data were tested and the results were analyzed. The images (of the test dataset) provided the performance metric values such as precision, recall, IoU, F1-score and test loss of 89.7%, 72.55%, 82.57%, 80.22% and 19.82%, respectively. Since the testing was performed in a real field condition, the proposed method provided slightly reduced results when compared with validation.

**Figure 6:**
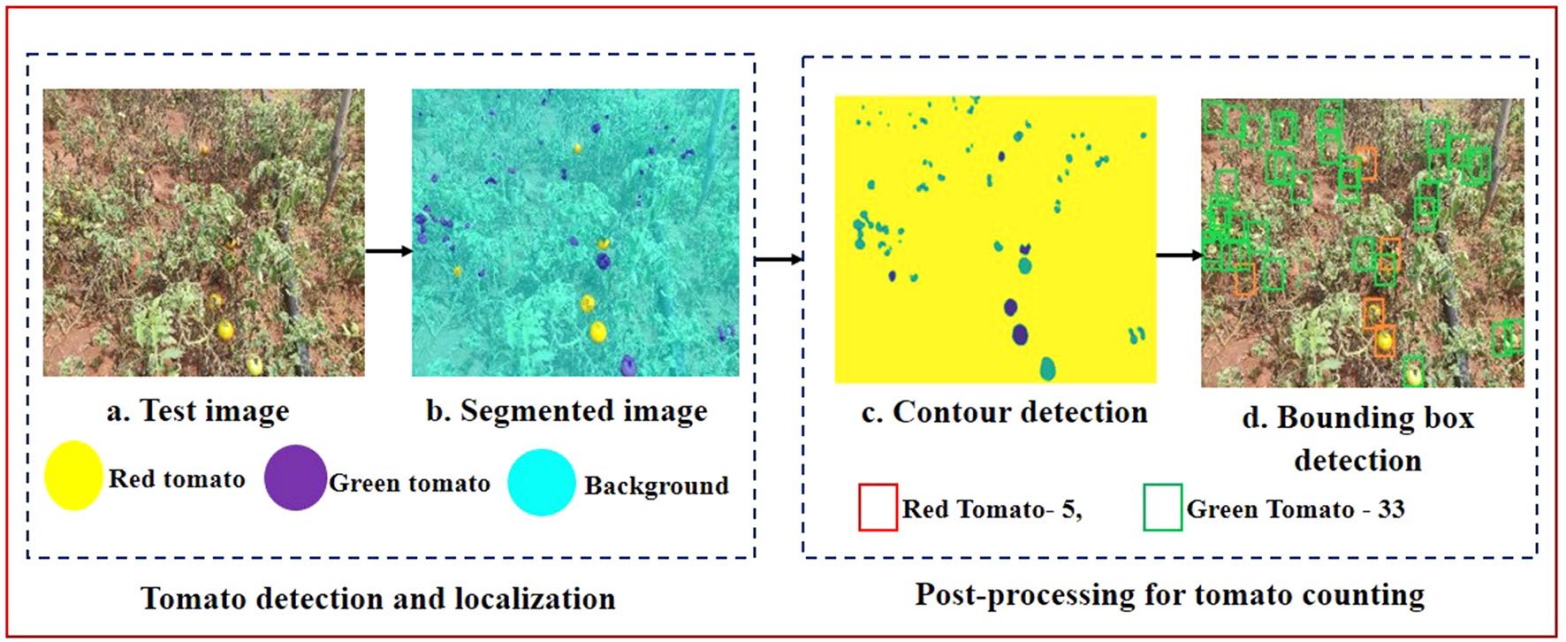
Test image tomato detection and counting using trained SegNet with VGG19 architecture.

**Figure 7:**
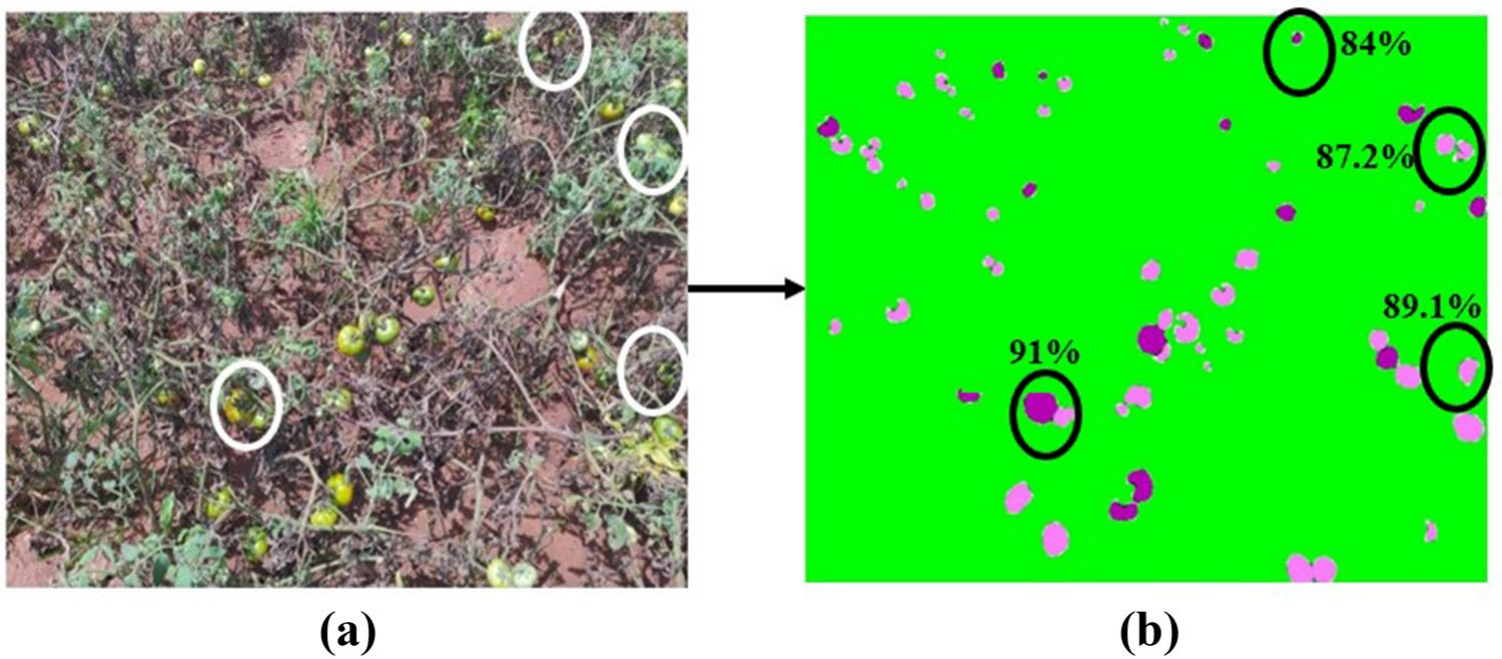
Prediction for occlusion (**a**) original test image, (**b**) predicted test image with precision (in %).

The segmented output is then subjected to post-processing in order to obtain the final yield of the tomato (as shown in Fig. 6). The closed region contours of the segmented output possess both the pixels of red and the green tomato. The bounding boxes are located at the region of contours using the coordinates. After many trials, an optimum threshold value of 0.5 was chosen for improving the prediction of the bounding box. The bounding box is eliminated, if its value is below the threshold inferring that there is no tomato in the specific region. Then the green and red tomatoes were classified based on the intensity value of the pixel. The proposed method provided better localization in an occluded environment. For example, in Fig. 7, the tomatoes that were occluded by the foliage, branch and fruits were marked by the white circle. The corresponding right side image demonstrates the percentage of tomato prediction (i.e., precision) by the proposed architecture. The amount of prediction is determined by the precision values. The detection rate was better (91%) if one fruit was occluded by another fruit by a smaller amount as shown in Fig. 7. On the other hand, if the fruit was occluded by other fruits, foliage and branches considerably, then the detection rate was reduced to 84%. The overall precision for an entire test dataset was 89.7%. The proposed method localizes the fruit satisfactorily due to its ability of pixel-wise classification to a particular class as well as fine-tuning the basic architecture by incorporating the three more layers. However, the precision value gets affected in the case of fully occluded fruits.

From the semantic segmented output, the tomatoes are counted using the bounding box drawn on each blob. The test data has given the tomato count of 4760 for the chosen field. It is compared with manual yield data collected from the farmers of Manapparai village, which is approximately 5000 tomatoes. The statistics of manual data collected from the farmers are given below:

- 25 cent (i.e., ¼ acre) ≃ 20 boxes of tomatoes.
- 1 box ≃ 20 kg of tomato.
- 1 kg of tomato ≃ 12 tomatoes.
- 25 cent field ≃ 5000 tomatoes.

Hence, the proposed method demonstrated the results closer to the real values.

To further ascertain its performance, the test results were compared with the recent research work of tomato YE using faster R-CNN trained with NVIDIA GeForce GTX 1080 graphics card by Mu et al.^28^. This work revealed an average precision value of 87.83% from the test dataset of 59 images. The proposed SegNet with VGG19 architecture provided an average precision value of 89.7% from the test dataset of 65 images. The attributed reason for obtaining an increased precision value (i.e., 89.7%) is due to the reduced number of FP (detection of tomato) thereby achieving a good learning capability.

In addition, the proposed method was compared with the modified Inception-ResNet-A module by Rahenemoonfar and Sheppard^30^. In this work, the architecture was trained with synthetic images, i.e., artificially the images were developed using red tomato blobs. The training was performed using the NVIDIA 980Ti GPU. In the testing phase, the work provided an accuracy of 91.03% for the test dataset comprising tomato images obtained from Google. Though the proposed method provided the test precision of 89.7%, the training, validation and testing were performed using the images obtained from an agricultural field.

## Conclusion

The DL-based semantic segmentation model using SegNet with VGG19 was customized to estimate the yield of the tomato crop. The original dataset of 123 tomato images was annotated and augmented in the preprocessing stage. The augmentation was done to avoid the overfitting problem during the training stage. After augmentation, the obtained dataset (of 672 images) was trained with the randomized weight and bias parameters. Using SGD back propagation algorithm, the error was minimized and optimized parameters were obtained. Using this trained network, the testing was done on the real (tomato field) data. The proposed model depicted reasonable results in the detection as well as counting of tomatoes. The results were compared with the other DL-based semantic segmentation architectures namely, SegNet with VGG16 and U-Net. A case study was also performed for testing the efficacy of SegNet with VGG19 architecture. It demonstrated the (test) precision, recall and F1-score values of 89.7%, 72.55% and 80.22%, respectively. The output of the proposed method provided insightful information about the next step for the farmers in terms of correct harvesting period, marketing as well as better cultivation practices. However, as VGG was used as the backbone network, execution time was one of the hurdles for implementation in real-time applications. But, it can be reduced by training the dataset with advanced DL-based semantic segmentation architectures such as YOLO, DeepLabV3+, etc., by which an augmented intelligent fruit YE model can be derived in the future.
